# Portal Vein and Superior Mesenteric Vein Thrombosis in a Patient With Antiphospholipid Syndrome and Chronic Hepatitis B

**DOI:** 10.7759/cureus.108125

**Published:** 2026-05-02

**Authors:** Lena Sayed Taha, Amal Katalo, Kiran Kumar, Shreya Nair, Shabaz Mohiuddin Ghulam, Arundeep Arora

**Affiliations:** 1 Medicine, Thumbay University Hospital, Ajman, ARE; 2 Internal Medicine, Thumbay University Hospital, Ajman, ARE; 3 College of Pharmacy, Gulf Medical University, Ajman, ARE; 4 Radiology, Thumbay University Hospital, Ajman, ARE

**Keywords:** antiphospholipid syndrome (apls), chronic hepatitis b (chb), mesenteric vein thrombosis, portal vein thrombosis (pvt), superior mesenteric vein thrombosis

## Abstract

A 56-year-old woman with chronic hepatitis B presented with a two-year history of left-sided cramping abdominal pain and constipation. Contrast-enhanced CT (CECT) of the abdomen revealed an eccentric filling defect in the main portal vein extending into the superior mesenteric vein, causing significant narrowing of the cross-sectional area, thereby suggesting thrombosis. A thin eccentric filling defect was also observed in the inferior mesenteric vein. Anticardiolipin antibody IgM was positive, while the remainder of the thrombophilia workup was unremarkable. A diagnosis of antiphospholipid syndrome (APS) was considered, and the patient was started on anticoagulants. Portal or mesenteric vein thrombosis should prompt immediate evaluation for underlying prothrombotic conditions such as APS.

## Introduction

Antiphospholipid syndrome (APS) is an autoimmune disorder characterized by arterial and/or venous thrombosis and/or adverse pregnancy outcomes, including recurrent fetal loss [1]. APS may occur as a primary (idiopathic) condition or in association with other autoimmune diseases, most commonly systemic lupus erythematosus (SLE) [2]. Thrombosis occurring at unusual sites, such as the portal or mesenteric veins, particularly in the absence of advanced liver disease, should raise suspicion of underlying prothrombotic conditions, including APS. The presentation of APS with concomitant portal and mesenteric venous thrombosis in the setting of chronic hepatitis B-related liver disease has been rarely reported [3].

## Case presentation

A 56-year-old woman presented with a two-year history of left-sided cramping abdominal pain and constipation. The pain was continuous, radiated to the back, and increased in intensity after meals, with a dull ache persisting between meals. She also reported chronic constipation and postprandial bloating. She denied fever, chills, nausea, vomiting, or weight loss. She was a known case of chronic hepatitis B and had been receiving entecavir 0.5 mg daily for the past five years. She denied a history of diabetes mellitus or other chronic medical illnesses. Her surgical history included a cesarean section and fibroidectomy without complications. On physical examination, her vital signs were within normal limits. Abdominal examination revealed a soft abdomen with nonspecific tenderness in the left lumbar region. There was no organomegaly, and bowel sounds were normal. Hernial orifices were intact. Cardiovascular and respiratory examinations were unremarkable. Laboratory investigations are summarized below in Table 1.

**Table 1:** Laboratory results PCR: polymerase chain reaction; DRVV: dilute Russell viper venom

Investigations	Result	Normal range	Unit
Hematology			
White blood cells	6,300	4.0 – 10.0	Cells/µL
Lymphocytes	61.6%		
Platelet count	137,000	150 – 410	Cells/µL
Glycemic control			
Fasting blood glucose	117	70 – 99	mg/dL
Postprandial blood glucose	172		mg/dL
Lipid profile			
Total cholesterol	277	<200, borderline: 200-239, high: >239	mg/dL
High-density lipoprotein (HDL) cholesterol	69	High risk: <40, low risk: 40-60, no risk: >60	mg/dL
Low-density lipoprotein (LDL) cholesterol	186	Up to 100	mg/dL
Liver function tests			
Aspartate aminotransferase (AST)	69	Up to 35	U/L
Alanine aminotransferase (ALT)	82	Up to 35	U/L
Gamma-glutamyl transferase (GGT)	227	38	U/L
Alkaline phosphatase (ALP)	119	35 – 104	U/L
Immunology/infectious screening			
Hepatitis B (HBeAb)	0.203 (reactive)	>1.000: non-reactive, <1.000: reactive	
Hepatitis B virus quantitative (PCR)	<20		IU/ML
Immunology/serology			
Cardiolipin antibody (IgM)	26.41 (low positive)	<12	MPL
Cardiolipin antibody (IgG)	8.21	<15	G.P.L
Hematology/coagulation			
Protein S, free	119	70 – 148% (male), 50-134% (female)	%
Protein C, activity	109.0	70.0 – 130.0	%
Molecular biology			
Factor V (Leiden) mutation (PCR)	Not detected		
Lupus anticoagulant screening			
PTT and mixing studies			
Activated partial thromboplastin time (APTT)	36.8	30.0 – 40.0	Sec.
Control	32		Sec.
PTT (test + control) 1:1	33.8		Sec.
DRVV screen time			
DRVV (test)	38.10	32.82 – 48.90	Sec.
DRVV (control)	35.50	32.82 – 48.90	Sec.
DRVV ratio	1.07	0.82 – 1.22	Sec.
Lupus anticoagulant			
Lupus anticoagulants (LAC)	Absent	Absent	
ANA-IFA (antinuclear antibodies)			
Antinuclear antibodies (ANA)	Negative	Negative	

**Figure 1:**
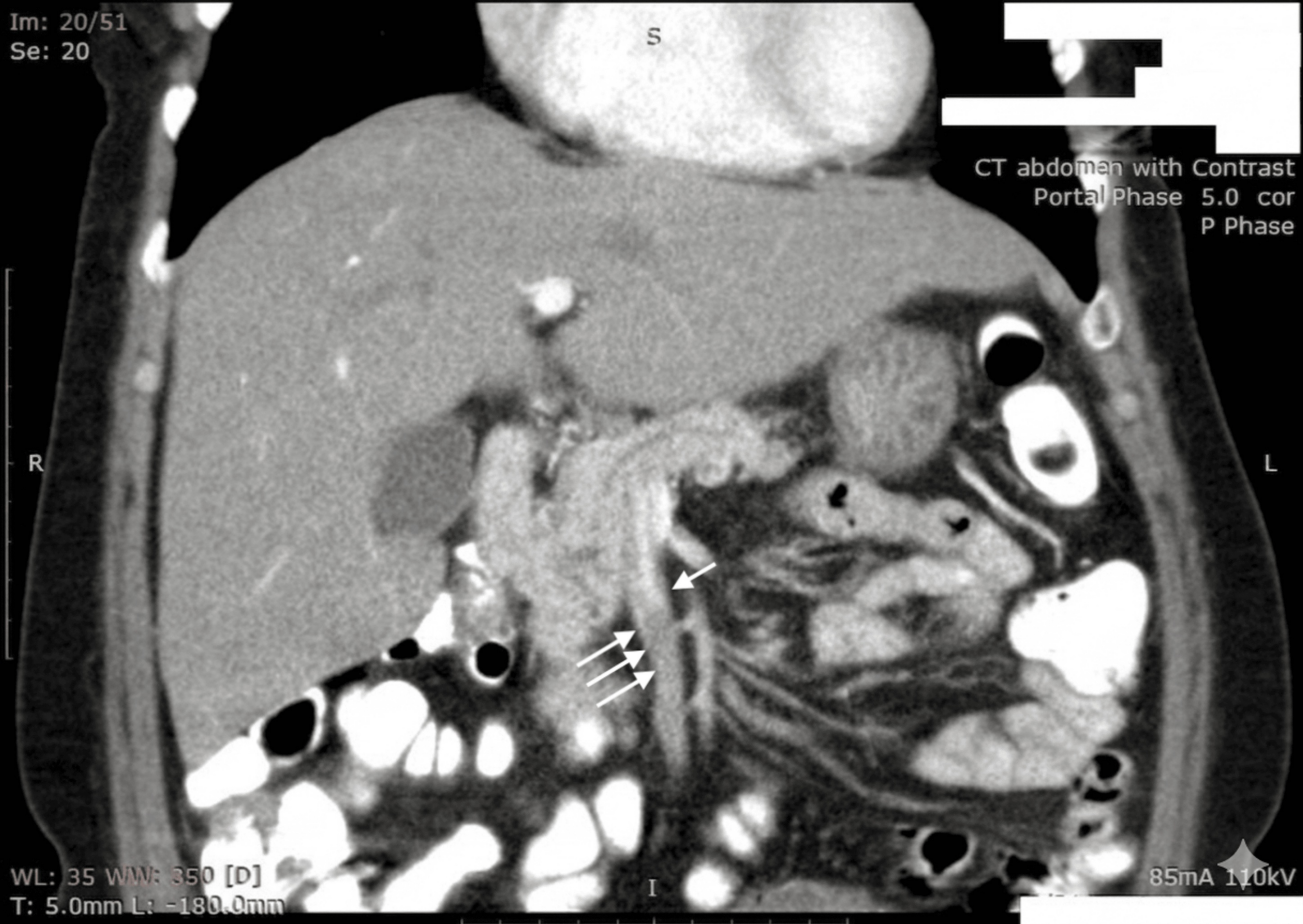
Contrast-enhanced CT abdomen (portal venous phase) The image shows filling defects in the middle and distal portions of the SMV (arrows), compared with the normal contrast enhancement of the proximal SMV (arrow) CT: computed tomography; SMV: superior mesenteric vein

Contrast-enhanced CT (CECT) of the abdomen demonstrated an eccentric filling defect in the main portal vein extending into the superior mesenteric vein, causing significant luminal narrowing, consistent with thrombosis (Figure 1). The adjacent mesenteric tributaries showed adequate contrast opacification. A thin eccentric filling defect was also noted in the inferior mesenteric vein. Hepatitis B viral DNA was not detectable.

Given the presence of portal and mesenteric vein thrombosis, the patient was evaluated for thrombophilia. Antinuclear antibodies and lupus anticoagulant were negative. Protein C, protein S, and factor V Leiden levels were within normal limits. Anticardiolipin antibody IgG was negative, while anticardiolipin antibody IgM was positive at 26.41 MPL. Activated partial thromboplastin time and dilute Russell viper venom time (DRVVT) were normal.

A diagnosis of antiphospholipid syndrome was made, and the patient was started on anticoagulation with subcutaneous enoxaparin sodium 60 mg twice daily. She was subsequently switched to oral rivaroxaban 15 mg twice daily for 21 days, followed by 20 mg once daily for long-term therapy. She was advised to continue entecavir indefinitely. At the three-month follow-up, the patient reported significant symptomatic improvement, with no evidence of any rivaroxaban-related adverse effects.

## Discussion

APS is characterized by antibodies directed against plasma proteins bound to anionic phospholipids [4]. Patients may present with venous or arterial thrombosis and adverse pregnancy outcomes, including recurrent fetal loss [1]. Thrombocytopenia may be observed on a complete blood count. APS may occur as a primary condition or develop secondarily in association with autoimmune diseases such as SLE and other rheumatologic disorders. Diagnosis is established using the International Consensus Criteria, which incorporate both clinical manifestations and laboratory findings [5]. Thrombosis at unusual sites, including the portal or mesenteric veins, should be evaluated immediately for underlying prothrombotic conditions [6]. As with venous thrombosis elsewhere, portal and mesenteric vein thrombosis may result from endothelial injury, venous stasis, or underlying thrombophilia [7,8]. Cirrhosis accounts for approximately 22-28% of cases, as demonstrated in extensive population-based autopsy studies [9].

Abdominal pain is the most common presenting symptom of portal or mesenteric vein thrombosis, often disproportionate to physical examination findings. Nonspecific symptoms such as nausea, vomiting, and diarrhea may occur with mesenteric vein involvement [10]. In patients with cirrhosis, portal vein thrombosis may be asymptomatic or present with abdominal pain and/or worsening liver disease. Chronic portal vein thrombosis often presents with features of portal hypertension. In contrast, our patient presented with a two-year history of abdominal pain and constipation without clinical evidence of portal hypertension.

Contrast-enhanced CT or MRI is recommended before initiating anticoagulation to confirm the diagnosis, differentiate bland from tumor thrombus, and assess the mesenteric vasculature for complications such as bowel ischemia, gangrene, or perforation [11]. Thrombophilia testing is recommended in patients without cirrhosis when no clear etiology is identified. In patients with cirrhosis, thrombophilia evaluation should be considered in those with a family history of thrombosis or thrombosis at unusual sites such as the hepatic veins [10].

Anticoagulation should be initiated as soon as possible in acute thrombosis unless bowel infarction or perforation is suspected, which requires urgent surgical intervention [12]. According to current clinical guidelines, including those from the American Society of Hematology (ASH) and the British Society of Haematology, the initiation of life-saving anticoagulation should not be delayed for thrombophilia testing, as results rarely alter initial management [12,13]. In patients with mesenteric venous thrombosis, anticoagulation improves recanalization rates, reduces the risk of bowel ischemia or the need for surgery, and improves survival [10]. Anticoagulation for chronic portal vein thrombosis is recommended in patients with cirrhosis who have underlying thrombophilia or with extension into the mesenteric veins with features of bowel ischemia [7]. Given the presence of chronic mesenteric vein thrombosis and early chronic liver disease, long-term anticoagulation was indicated in our patient. This case was unique due to the coexistence of APS and chronic hepatitis B with early chronic liver disease, resulting in portal and mesenteric vein thrombosis.

## Conclusions

This case report highlights APS as an important and often underrecognized cause of splanchnic venous thrombosis, especially in patients without advanced cirrhosis. Clinicians should include splanchnic venous thrombosis in the differential diagnosis for patients presenting with chronic abdominal pain that is out of proportion to physical examination findings. Early cross-sectional imaging is essential for timely diagnosis. Furthermore, the coexistence of APS and chronic hepatitis B in this patient underscores the importance of performing a comprehensive thrombophilia evaluation, even when an underlying liver disease is already present, to enable prompt anticoagulation and reduce the risk of life-threatening bowel ischemia.
